# Prospective cohort study of basivertebral nerve ablation for chronic low back pain in a real-world setting: 12 months follow-up

**DOI:** 10.1016/j.inpm.2024.100446

**Published:** 2024-11-25

**Authors:** William Schnapp, Moacir Schnapp, Jonathan Gottlieb, Lucien C. Alexandre, Kenneth Martiatu, Gaëtan J.-R. Delcroix

**Affiliations:** a NeuroSpine & Pain Center, Key West, FL, USA; b Minimally Invasive Spine Center of South Florida, Miami, FL, USA; c Nova Southeastern University, College of Allopathic Medicine, Fort Lauderdale, FL, USA; d Neuroscience Associates, Key West, FL, USA

**Keywords:** Chronic low back pain, Basivertebral nerve ablation, Radiofrequency, Endplate degeneration, Modic changes, Community setting, Independent study

## Abstract

**Background:**

The basivertebral nerve, which densely supplies the vertebral endplates, is a potential source of chronic low back pain transmission in patients with Modic changes. Basivertebral nerve ablation (BVNA), a minimally invasive procedure, aims to disrupt this pain signaling.

**Objectives:**

In this study, we investigated BVNA's effectiveness in treatment of vertebrogenic low back pain and we followed patients for 12 months to assess long-term effectiveness.

**Study design:**

Single group prospective cohort study (ClinicalTrials.gov NCT05692440).

**Setting:**

Single-center, community private practice.

**Methods:**

Thirty-five patients were treated with the INTRACEPT® device (Boston Scientific, MA, USA). Thirty-one patients completed Oswestry Disability Index (ODI), Visual Analog Scale (VAS), SF-36 Physical Component Summary (PCS), and SF-36 Mental Component Summary (MCS) at baseline and follow-up visits up to 12 months.

**Results:**

The average age of the 31 patients was 73.0 ± 6.34 years and 71.0 % of the population was male (N=22)) at baseline. All four self-reported outcomes (ODI, VAS, SF-36 PCS, and MCS) showed statistically and clinically significant improvements from baseline through 12 months (all p < 0.001, with the exception of the SF-36 MCS at 1 month, p = 0.165). Overall, 67.7 % of patients demonstrated ODI improvements above the minimal clinically important difference (decrease of at least 15 points) and 77.4 % of patients demonstrated a decrease on the VAS above the minimal clinically important difference (≥2 cm reduction) at 12 months.

**Limitations:**

Limitations of the study include the lack of a control group and potentially unintentional bias in patient selection.

**Conclusions:**

BVNA demonstrates potential as an effective and minimally invasive treatment for chronic low back pain in a real-world patient cohort where substantial improvements were observed. These results align with those seen in previous randomized controlled trials (RCTs) and industry-funded studies of BVNA.

## 1 Background

Few conditions have a greater impact on quality of life, worldwide productivity, and economic burden as chronic low back pain (CLBP) [1]. There is significant guideline discordance in diagnosing and treating CLBP [2].

Among the standard procedures commonly available in the treatment arsenal for CLBP, lumbar fusions remain debated and controversial, and discography to select such patients has also been criticized [3]. A 6-month course of conservative treatment is generally recommended before considering fusion. This includes physical therapy, exercise, and non-opioid pain management strategies. However, many patients who undergo lumbar fusions for CLBP, even when ideally selected, do not appreciate a substantial improvement in their symptomatology [[4], [5], [6], [7], [8]].

Basivertebral nerve ablation (BVNA) is a relatively new spinal procedure that emerged for the treatment of CLBP in patients with Modic changes (Type 1 or 2). BVNA has been proven effective in several studies [9,10], and it was recently described by McCormick et al. that the utilization of conservative care, opioids, lumbosacral spinal injections, lumbosacral radiofrequency ablation, and lumbar fusion rate were substantially reduced through 5 years post-BVNA compared to baseline. These findings emphasize the possible benefit of this procedure to reduce the financial burden of CLBP and healthcare utilization [11]. In the USA, two RCTs and two prospective cohort studies of BVNA have already driven changes in the treatment algorithm for CLBP [9]. Still lacking, however, are non-industry funded real-world results on this procedure.

Given these important inflexion points in CLBP treatment, we performed this study to report on the effectiveness of BVNA in a non-industry funded real-world setting.

## 2 Methods

***Study design.*** This is a single group prospective cohort study (ClinicalTrials.gov NCT05692440). This study was HIPPA compliant and conducted with institutional review board approval and participant informed consent. Consecutive participants meeting criteria were enrolled from a single center private practice between May 2021 and February 2023. All data was recorded in Medrio electronic data capture software (Medrio, San Francisco, CA) by a clinical research associate (KM).

***Participants.*** The inclusion/exclusion criteria are listed in Table 1.

**Table 1:** Inclusion and exclusion criteria. In practice, all the patients we included had CLBP for more than 5 years as mentioned in the result section.

Inclusion Criteria	Exclusion Criteria
Adult patients ≥18 years of age.	Patients with severe cardiac or pulmonary disease.
Patients who have experienced chronic low back pain for ≥6 months.	Patients with active systemic infection or localized infection in the treatment area.
Patients who have not responded to at least 6 months of conservative care.	
Patients with Modic type 1 or 2 changes.	
Patients with pain upon flexion during physical examination.	

**Fig. 1:**
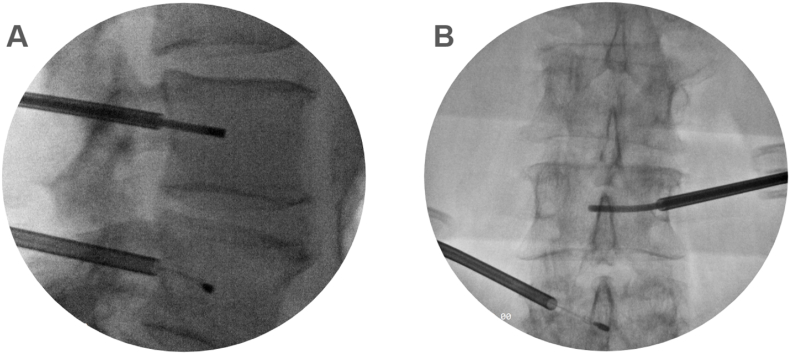
Figure A depicts a lateral view of a BVNA procedure at the L3 and L4 level. At L4, the probe has been deployed and ablation is in process. During the ablation at L4, the J-stylet is positioned in the L3 vertebral body in preparation for subsequent ablation at L3. BVNA: basivertebral nerve ablation.

***Study intervention.*** BVNA was performed by a single surgeon (WS) utilizing the INTRACEPT® device (Boston Scientific, MA, USA) as previously described by Fischgrund et al. [12]: the procedure is performed unilaterally with the patient in a prone position; either general or conscious sedation is administered. Using standard anatomic landmarks, the location of the entry pedicle at each level to be treated is determined and marked. Under fluoroscopic guidance, an introducer cannula is advanced through the pedicle until the trocar just breaches the posterior vertebral wall. The introducer trocar is exchanged with a smaller plastic cannula/curved nitinol stylet assembly, which facilitates the creation of a curved path from the posterior wall to the pre-determined target located at the terminus of the BVN, located near the center of the vertebral body. Finally, the curved nitinol stylet is removed and an RF probe is introduced and positioned at the terminus of the BVN. See Fig. 1 for details. The bipolar RF probe is activated and the temperature at the tip is maintained at a constant 85 °C for 15 min. No assessment of targeting success was performed on patients that did not have improvement in CLBP post BVNA.

**Fig. 2:**
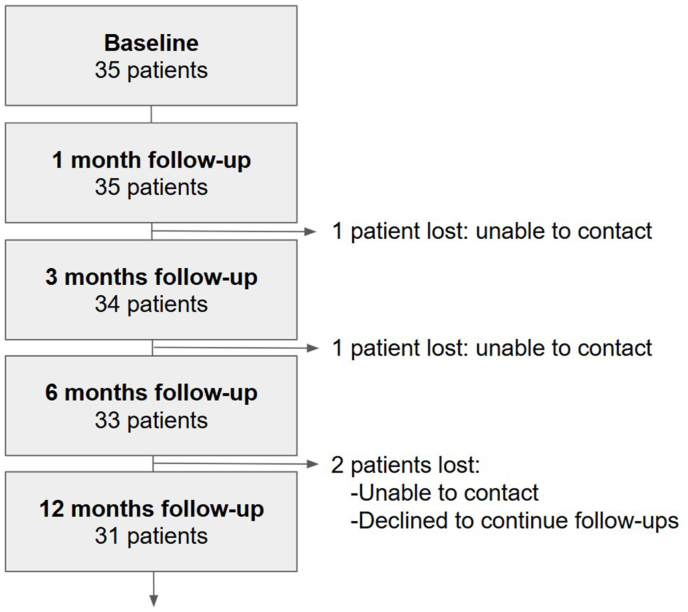
Flow diagram of patients lost during follow-up.

***Outcome measures.*** Evaluations were performed at baseline, and at 1, 3, 6, and 12 months post-BVNA and reported only for the patients that completed their 12 months assessment. The 4 patients lost to follow-ups (see Fig. 2) were not included in the data reported, except for the worst-case percentage calculations presented in the results section. Baseline evaluations were performed between 1 and 3 weeks before BVNA. Clinical outcomes were measured using the Oswestry Disability Index (ODI) and the Visual Analog Scale (VAS). ODI categories were defined as per Fairbank et al. [13]: 0 %–20 % (minimal disability): the patient can cope with most living activities. Usually, no treatment is indicated apart from advice on lifting, sitting, and exercise. 21%–40 % (moderate disability): the patient experiences more pain and difficulty with sitting, lifting, and standing. Travel and social life are more difficult and they may be disabled from work. Personal care, sexual activity, and sleeping are not grossly affected and the patient can usually be managed by conservative means. 41%–60 % (severe disability): pain remains the main problem in this group but activities of daily living are affected. These patients require a detailed investigation. 61%–80 % (crippled): back pain impinges on all aspects of the patient's life. Positive intervention is required. 81%–100 %: these patients are either bed-bound or exaggerating their symptoms. Changes in physical function and mental health were assessed using the Short Form Health Survey (SF-36) Physical Component Summary (PCS) and Mental Component Summary (MCS) scales.

**Fig. 3:**
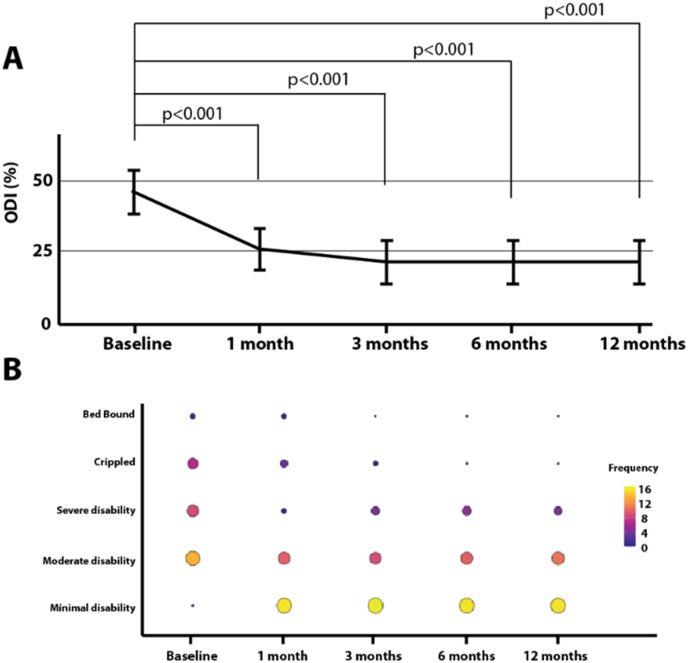
Oswestry Disability Index (ODI). ODI results revealed a significant reduction from baseline in the subject's pain impact (A) but also a reduction in the number of individuals reporting being crippled or bed-bound (B). ODI categories were defined as follows: 0 %–20 % (minimal disability): the patient can cope with most living activities. Usually, no treatment is indicated apart from advice on lifting, sitting, and exercise. 21%–40 % (moderate disability): the patient experiences more pain and difficulty with sitting, lifting, and standing. Travel and social life are more difficult and they may be disabled from work. Personal care, sexual activity, and sleeping are not grossly affected and the patient can usually be managed by conservative means. 41%–60 % (severe disability): pain remains the main problem in this group but activities of daily living are affected. These patients require a detailed investigation. 61%–80 % (crippled): back pain impinges on all aspects of the patient's life. Positive intervention is required. 81%–100 %: these patients are either bed-bound or exaggerating their symptoms. Figure A depicts the mean and 95 % confidence interval plot of ODI scores by visits.

**Fig. 4:**
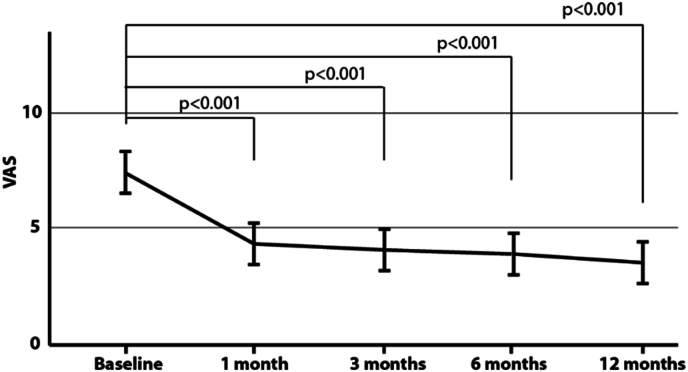
Visual Analog Scale (VAS). We observed a significant improvement between VAS scores at one-month, three-month, six-month and 12-months follow-up visits compared to baseline (*p* < 0.05). The figure depicts the mean and 95 % confidence interval plot of VAS scores by visits.

**Fig. 5:**
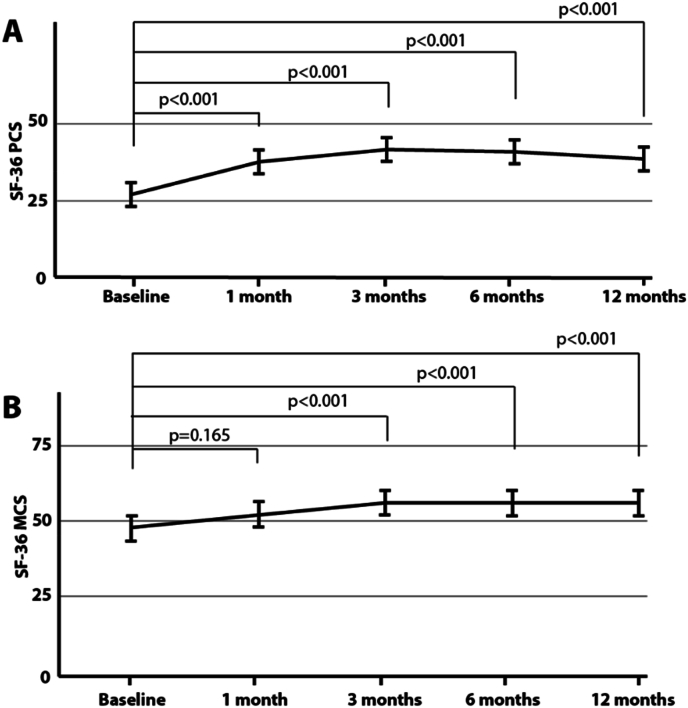
SF-36. We observed a significant improvement between SF-36 PCS scores at one-month, three-month, six-month and 12-months follow-up visits compared to baseline (all *p* < 0.001) (A). We observed a significant improvement between SF-36 MCS scores at three-month, six-month and 12-months follow-up visits compared to baseline (*p* = 0.165 at 1 month and p < 0.001 afterwards) (B). The figure depicts the mean and 95 % Confidence Interval Plot of SF-36 T-Scores by visits.

***Statistical analysis.*** Data are reported as mean ± standard deviation unless otherwise specified. Fig. 3, Fig. 4, Fig. 5 depicts the mean and 95 % confidence interval plot by visits. Linear mixed models with clustered standard errors were used to look for differences across visits; where the fixed effects were visits and the random effect was the subject. Results were adjusted for age, gender, baseline back pain on average over the past 7 days, and how long the patient had lower back pain. For post-model comparisons, we used a Tukey-HSD test. For the SF-36 Physical and Mental Functioning measures, we used T-Scores which have a mean of 50 and a standard deviation of 10. Statistical significance was found at p < 0.05. R 4.3.2, and STATA 18.0 were used in all data analyses. We also reported on the percentage of patients exhibiting the Minimal Clinically Important Differences (MCID) for VAS (≥2 cm improvement) and ODI (≥15-point improvement) [14]. The percentage of patients experiencing a decrease of VAS superior or equal to 50 % is also reported.

## 3 Results

***Demographics and baselines.*** 35 patients were enrolled, and 31 completed the 12 month follow-up (89 % retention, see Fig. 2). The average age of the 31 patients was 73.0 ± 6.34 years and 71.0 % of the population was male (N=22)) at baseline. The majority of them (64.5 %) had low back pain for more than 5 years (N = 20). Our study had less stringent inclusion criteria than previously published studies [14]. Patients with radicular pain were not excluded from this study (1 patient). Patients were not screened for osteoporosis. The minimum VAS score at baseline was 4 cm and the minimum ODI at baseline was 24 %. 5 patients had a history of spinal fusion surgery and 15 had prior lumbar medial branch RFA. 10 out of 35 (28.6 %) were prescribed opioids. The majority of patients received BVNA at 2 or 3 levels, more specifically as follows.; L2-L3: 2 patients; L3-L4: 2 patients; L4-L5: 9 patients; L5-S1: 1 patient; L1-L2-L3: 2 patients; L2-L3-L4: 1 patient; L3-L4-L5: 13 patients; L4-L5-S1: 1 patient. Single level treatment was performed adjacent to hardware on 4 patients: L2: 1 patient; L4: 1 patient; L5: 2 patients.

***Oswestry Disability Index (ODI*).** ODI at baseline was 46.7 ± 15.8 % and decreased to 21.5 ± 16.9 % at 12 months (p < 0.001). More specifically, ODI declined by 20.7 points [95 % CI: 11.8, 29.5] at 1 month, 25.0 points [95 % CI: 16.3, 33.8] at 3 months, 24.7 points [95 % CI: 15.8, 33.5] at 6 months, and 25.2 points [95 % CI:16.3, 34.0] at 12 months from baseline, Fig. 3A. ODI category analysis revealed a significant reduction in the number of patients reporting being bed bound, crippled, or severely disabled from baseline through 12 months, Fig. 3B. Overall, 67.7 % of the patients demonstrated ODI improvements above the MCID (decrease of 15 points). If we consider the scenario in which patients lost to follow-up (N=4) would not have had any improvement beyond MCID at 12 months, the percentage of patients with ODI benefits beyond MCID is reduced to 60.0 % of the patient population. A summary of the data and their statistics is presented in Table 2.

**Table 2:** Outcomes summary. Values are expressed as mean with standard deviation in brackets. For the ODI categories, the first number refers to the number of patients, and the number in parentheses to the corresponding percentage out of the 31 patients. P values are presented for each timepoint compared to baseline.

Variable	Baseline	1 month	3 months	6 months	12 months
**ODI (%)**	46.7 (15.8)	26.0 (22.6) p < 0.001	21.7 (17.0) p < 0.001	22.0 (17.1) p < 0.001	21.5 (16.9) p < 0.001
Minimal Disability	0 (0 %)	16 (51.6 %)	17 (54.8 %)	16 (51.6 %)	16 (51.6 %)
Moderate Disability	14 (45.2 %)	10 (32.3 %)	9 (29.0 %)	10 (32.3 %)	11 (35.5 %)
Severe Disability	9 (29.0 %)	1 (3.2 %)	4 (12.9 %)	5 (16.1 %)	4 (12.9 %)
Crippled	7 (22.6 %)	3 (9.7 %)	1 (3.2 %)	0 (0 %)	0 (0 %)
Bed Bound	1 (3.2 %)	1 (3.2 %)	0 (0 %)	0 (0 %)	0 (0 %)
**VAS Pain Scale**	7.4 (1.2)	4.3 (2.5) p < 0.001	4.1 (2.6) p < 0.001	3.9 (2.2) p < 0.001	3.5 (2.4) p < 0.001
**SF-36 Physical Functioning**	26.8 (5.3)	38.0 (11.7) p < 0.001	42.3 (11.0) p < 0.001	40.9 (12.1) p < 0.001	38.8 (10.4) p < 0.001
**SF-36 Mental Functioning**	47.4 (11.6)	51.6 (10.0) p = 0.165	56.1 (9.3) p < 0.001	55.8 (9.4) p < 0.001	56.4 (7.8) p < 0.001

***Visual Analog Scale (VAS).*** VAS at baseline was 7.4 ± 1.2 cm and decreased to 3.5 ± 2.4 cm at 12 months (p < 0.001). More specifically, VAS was reduced by 3.1 cm [95 % CI: 1.7, 4.4] at 1 month, 3.3 cm [95 % CI: 1.9, 4.6] at 3 months, 3.5 cm [95 % CI: 2.1, 4.8] at 6 months, and 3.9 cm [95 % CI: 2.5, 5.2] at 12 months from baseline, Fig. 4. Overall, 77.4 % of patients demonstrated a decrease onVAS above the MCID (at least 2 cm reduction) at 12 months. If we consider the scenario in which patients lost to follow-up (N=4) would not have had any significant benefit at 12 months, the percentage of patients that demonstrated a VAS improvement above MCID would be 68.6 % of the patient's population. 54.8 % of patients demonstrated a decrease on VAS of at least 50 % at 12 months (48.6 % if we include the 4 patients lost to follow-up).

**SF-36 PCS.** SF-36 PCS at baseline was 26.8 ± 5.3 % and improved to 38.8 ± 10.4 % at 12 months (p < 0.001). More specifically, physical functioning improved by 11.2 [95 % CI: 5.6, 16.7] at 1 month, 15.5 [95 % CI: 9.9, 21.0] at 3 months, 14.1 [95 % CI: 8.5, 19.7] at 6 months, and 12.0 [95 % CI: 6.4, 17.5] at 12 months from baseline, Fig. 5A.

**SF-36 MCS.** SF-36 MCS at baseline was 47.4 ± 11.6 % and improved to 56.4 ± 7.8 % at 12 months (p < 0.001). More specifically, mental functioning improved by 4.2 [95 % CI: 0.9, 9.4] at 1 month, 8.7 [95 % CI: 3.5, 13.9] at 3 months, 8.4 [95 % CI: 3.1, 13.5] at 6 months, and 9.0 [95 % CI: 3.7, 14.2] at 12 months from baseline, Fig. 5B.

## 4 Discussion

The 12-month follow-up data presented in this manuscript demonstrates the effectiveness of BVNA in alleviating CLBP in older patients with Modic Type 1 or 2 changes. Improvements were statistically significant, clinically meaningful, and involved improvement in multiple domains including pain and physical function.

In their meta-analysis of former BVNA studies, Conger et al. reported 64 % of patients with ≥50 % VAS pain relief and 75 % of patients with a ≥15-point Oswestry Disability Index score improvement at 12 months [15]. In our study, 67.7 % of the patients demonstrated ODI improvements ≥15 points. If we consider the most conservative analysis in which patients lost to follow-up (4 patients) would not have had any significant benefit at 12 months, the percentage of patients with ODI benefits beyond MCID is calculated at 60.0 % of the patient population. In our study, 54.8 % of patients demonstrated a decrease on VAS of at least 50 % at 12 months, which recalculates at 48.6 % in the most conservative analysis after including the 4 patients lost to follow-up. Our results are therefore within the range of results reported in the meta-analysis by Conger et al. [15].

Our results are only slightly lower than the average ODI and VAS outcomes observed in the aforementioned meta-analysis. Potential reasons for this could include the less stringent real-world inclusion criteria. This includes patients with prior spinal fusion, and inclusion of patients with lower baseline levels of pain and functional impairment. In addition, our real-world study included much older patients as a function of non-coverage by most commercial insurance payers at the time of patient enrollment. It is also possible that patients of more advanced age have multiple pain generators. Despite this slight reduction in effectiveness compared to the aforementioned meta-analysis [14], the use of BVNA in our patient population still produced statistically and clinically significant pain reduction in a cohort of patients who would likely have had no other durable treatment available.

Limitations of the study include the lack of a control group and potentially unintentional bias in patient selection. Post-operative image surveillance was not performed in our study. Patients did not report any symptoms of compression fractures, an adverse event recently reported by Fogel et al. in some patients post-BVNA [16].

This study provides independent evidence that BVNA performed with the INTRACEPT® device in a community practice on an older patient population improves pain, physical function, and mental function for at least 12 months.

## 5 Conclusions

In this longitudinal series performed in a community practice setting on an older patient population, we observed statistically significant improvements for up to 12 months using ODI, VAS, and SF-36 scales. Importantly, 67.7 % of patients demonstrated ODI improvements above the MCID (decrease of 15 points) and 77.4 % of patients demonstrated a decrease on VAS above the MCID (at least 2 cm reduction) at 12 months.

## Statement of informed consent

This study was conducted in accordance with the ethical guidelines of the WCG IRB's Institutional Review Board (IRB). The study protocol and consent procedures were approved by the IRB (Protocol Reference Number: 20223348). Written informed consent was obtained from all participants prior to their involvement in the study and the study was registered with ClinicalTrials.gov (Ref #NCT05692440).

## Declaration of competing interest

The authors declare that they have no known competing financial interests or personal relationships that could have appeared to influence the work reported in this paper.
